# Affective dimensions of pain and region -specific involvement of nitric oxide in the development of empathic hyperalgesia

**DOI:** 10.1038/s41598-020-66930-w

**Published:** 2020-06-23

**Authors:** Fatemeh Mohammadi, Kristi Anne Kohlmeier, Sajad Jeddi, Meysam Ahmadi-Zeidabadi, Mohammad Shabani

**Affiliations:** 1 Intracellular Recording Lab, Kerman Neuroscience Research Center, Neuropharmacology Institute, Kerman University of Medical Sciences, Kerman, Iran; 2 Department of Drug Design and Pharmacology, Faculty of Health and Medical Sciences, University of Copenhagen, Copenhagen, 2100 Denmark; 3 Endocrine Physiology Research Center, Research Institute for Endocrine Sciences, Shahid Beheshti University of Medical Sciences, Tehran, Iran

**Keywords:** Neuroscience, Psychology

## Abstract

Empathy for pain depends on the ability to feel, recognize, comprehend and share painful emotional conditions of others. In this study, we investigated the role of NO in a rat model of empathic pain. Pain was socially transferred from the sibling demonstrator (SD) who experienced five formalin injection to the naïve sibling observer (SO) through observation. SO rats received L-NAME (a nonspecific NO synthase inhibitor) or L-arginine (a precursor of NO) prior to observing the SD. Nociception, and concentrations of NO metabolites (NOx) in the serum, left and right hippocampus, prefrontal cortex, and cerebellum were evaluated. Nociceptive responses were significantly increased in the pain-observing groups. NOx levels measured 24 h after the last pain observation using the Griess method, were indicative of NOx concentration decreases and increases in the left hippocampus and cerebellum, respectively. There was an increase in tissue concentration of NOx in cerebellum and prefrontal cortex in both pain and observer groups 7 days after the fifth formalin injection. Our results suggest that NO is involved in development of empathic hyperalgesia, and observation of sibling’s pain can change NO metabolites in different brain regions in observer rats.

## Introduction

Pain is perceived not only by personal experience, but also vicariously through social communication and interaction^1–5^. It is believed that feeling the pain of others depends on empathy, which is an ability to feel, recognize, comprehend and share the painful emotional condition of others^5–9^. Experimental human studies have shown that empathy for pain may lead to enhanced pain perception and evoked emotional responses in the observer^10^. While it had previously been thought that empathy is unique to human beings^7,11^, recent studies have led to the interpretation that empathy can be expressed by animals^12,13^. In a seminal study, mice witnessing the pain of a cagemate demonstrated hyperalgesia, suggesting not only empathy, but also that the mechanism of sensitization of empathetic pain were generalized^14^. Upon witnessing pain of a cagemate following bee venom injection naïve observer rats exhibited hypersensitivity as well as mechanical pain in both sides of the paws^15^. Another study indicated that mice witnessing cagemates with peripheral nerve injury exhibit enhancement of hyperalgesia in the acetic acid–induced writhing test^16^. Further, as the tendency of rodents to help other conspecifics in harmful situations is based on empathetic responses^12^, the demonstration that rats can exhibit helping behaviors in response to observation of pain in a conspecific provides further suggestion of empathetic behavior in rats^17,18^. Empathetic responses to pain do seem to rely on sensory discrimination as while mice with hind paw swelling induced by saline injection did not trigger attention signals in conspecifics, pain signals were transferred from a mouse experiencing pain^19^. Further, it has been noted that empathetic responses in conspecifics rely on co habitation for a duration exceeding two weeks^2,3,5^. When taken together, the data suggest that empathetic behavior triggered by painful experiences in conspecifics should be considered part of the emotional responses of rodents^6^.

Human neuroimaging data and experiments conducted in rodent models of empathy show that the observation of another person in pain causes the activation of neural networks in anterior cingulate cortex (ACC), anterior insular (AI), amygdala, mediodorsal thalamic nuclei^20–25^ and cerebellum^26,27^. Interestingly, area 24 in the rat ACC which is a part of medial prefrontal cortex (mPFC)^28^ contains mirror neurons that respond to both first-hand pain experiences and observations of another rat in pain^29^. These neurons encode the others’ pain similar to first-hand pain experience. Although the hippocampus is not considered a primary region in the network of brain regions involved in pain processing, and it has not been widely studied for its role in empathy, the known functions of the hippocampus within the limbic system suggest it could be importantly involved in empathetic pain. Consistent with this, rodent models of neuropathic pain showed differences in hippocampal-dependent behaviors and alterations in synaptic plasticity and hippocampal neurogenesis^30^. Interestingly, reductions in hippocampal volume have been seen in patients with chronic pain, which could contribute to emotional declines observed in chronic pain patients^30^. Further, while the study group was small, patients with hippocampal dysfunction exhibited reduced cognitive and emotional empathy when compared to controls^31^. As the hippocampus is crucial in learning and memory, and a major player in the emotion-controlling limbic system, and alterations in its functioning have been seen upon the pain experience and in those exhibiting deficits in empathetic responding^30^, it could also play a role in empathetic pain. Strong lateralization of hippocampal function has been demonstrated (for review see Jordan.,)^32^. Based on results of our study, expression of empathetic pain could be lateralized with the left hippocampus exhibiting a more predominant role.

While neural regions mediating emotional pain have been recognized, the underlying molecular mechanisms of empathic pain still remain poorly understood. The nitric oxide (NO) system has been shown to be involved in pain transmission (for review see, Cury *et al*.)^33^. Higher levels of NO were found in the cerebrospinal fluid (CSF) of patients with chronic pain^34^. Higher levels of NO metabolites (nitrate + nitrite = NOx) have also been detected in patients with chronic orofacial pain when compared to those in controls^35^. Animal models have determined that NO plays a crucial role in behaviors exhibiting alterations in pain thresholds, including spinal cord hyperalgesia (for review see, Cury *et al*. 2011)^33^. Further, an animal model of traumatic brain injury showed that NO2^−^/NO3^−^ levels in four different brain regions of the rats (cortex, cerebellum, hippocampus and brainstem) were uniformly reduced after head injury^36^.

While the molecular mechanisms of empathetic pain have not been elucidated, NO is a free radical^37^ synthesized from L-arginine (L-Arg; a precursor of NO) by three known NO synthases (neuronal (nNOS), endothelial (eNOS) and inducible (iNOS)^38–40^, and the gene for nNOS (nitric oxide synthesis 1) has been shown to play an essential role in social behavior such as empathy^41^.

Using rodent empathic pain models, we previously demonstrated that NO exerts a modulatory effect on empathy-induced changes in nociception, motor function and spatial memory^42^. Further, we have shown that alterations in NO synthesis altered nociception in observers of conspecifics receiving pain^42^. However, the modulating effect of NO in empathic pain, as well as the central pathways and neural regions involved in this activity were unexplored.

Since NO plays an important role in pain^43^ and we have shown a role for NO in empathic pain^42^, the aim of this study was to further explore this role in brain regions involved in empathic pain. As empathy in humans can be exacerbated by observing experience of chronic pain, we utilized a rat model of empathetic pain by sub-chronic witnessing of a pain experience of a sibling which was somewhat similar to human life. Pain empathy was evaluated by determination of nociception to painful thermal stimuli using two different behavioral tests. We evaluated whether empathic pain affected production of NO in the serum, and four regions of the rat brain relevant to empathy. To this end, NOx concentrations were assayed in the right and left hippocampus, cerebellum, and prefrontal cortex, 24 h and 7days after observation of the sibling in pain with the aim to pinpoint significant regional differences in NO production.

## Result

### Latency to show a nociceptive response in Hot plate test

**Figure 1:**
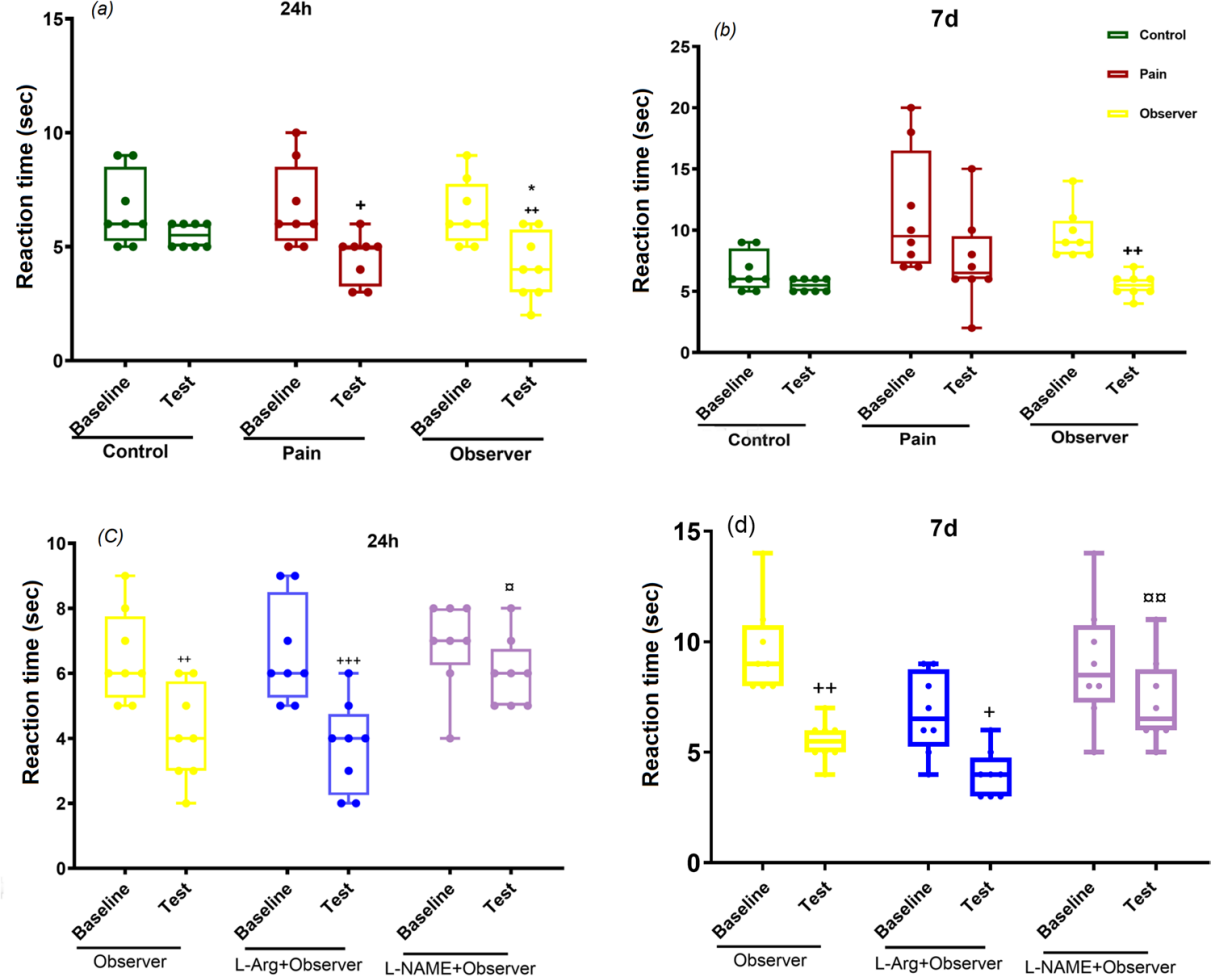
Effect of observation of sibling’s pain on reaction time in hot plate test in observer rats, 24 h (**a**) and 7d (**c**) after the last observation. Effects of L-Arg or L-NAME on the reaction time of observers (**b,d**). Results are shown as median with interquartile range (boxes) and maxima/minima (whiskers). ^**+**^p < 0.05, ^**++**^p < 0.01, ^**+++**^p < 0.001, *p < 0.05, **¤¤**p < 0.01. ^**+**^, *and ¤significantly different from the baseline, control and observer pretreated with L-Arg groups, respectively.

In the hot plate test, a nociceptive response latency was significantly reduced in all pain and observer groups at both 24 h and 7 day following the last pain observation in comparison to the pre-observation period (24 h: Fig. 1a, baseline & test: F_(1,42)_ = 29.1, p < 0.01, between groups: F_(2,42)_ = 3.5, P = 0.038; Fig. 1c, baseline & test: F_(1,42)_ = 21.6, p < 0.001, between groups: F_(2,42)_ = 0.4, P = 0.6). These data suggest induction of hyperalgesia in the observer group is likely due to experience of empathetic pain. A role of NO in hyperalgesia was suggested by findings that reduction in response time in the hot plate test was significantly attenuated in the L-arginine group in comparison to baseline, whereas, the reduction in response latency was significantly smaller in presence of L-NAME (7d: Fig. 1b, baseline & test: F_(1,42)_ = 29.7, p < 0.001, between groups: F_(2,42)_ = 3.3, P = 0.04; Fig. 1d, baseline & test: F_(1,42)_ = 28.3, p < 0.01, between groups: F_(2,42)_ = 9.6, p = 0.004). These data provide support of our earlier findings^42^ and indicate that NO plays a role in empathic pain signaling.

### Latency to show a nociceptive response in Tail flick test

**Figure 2:**
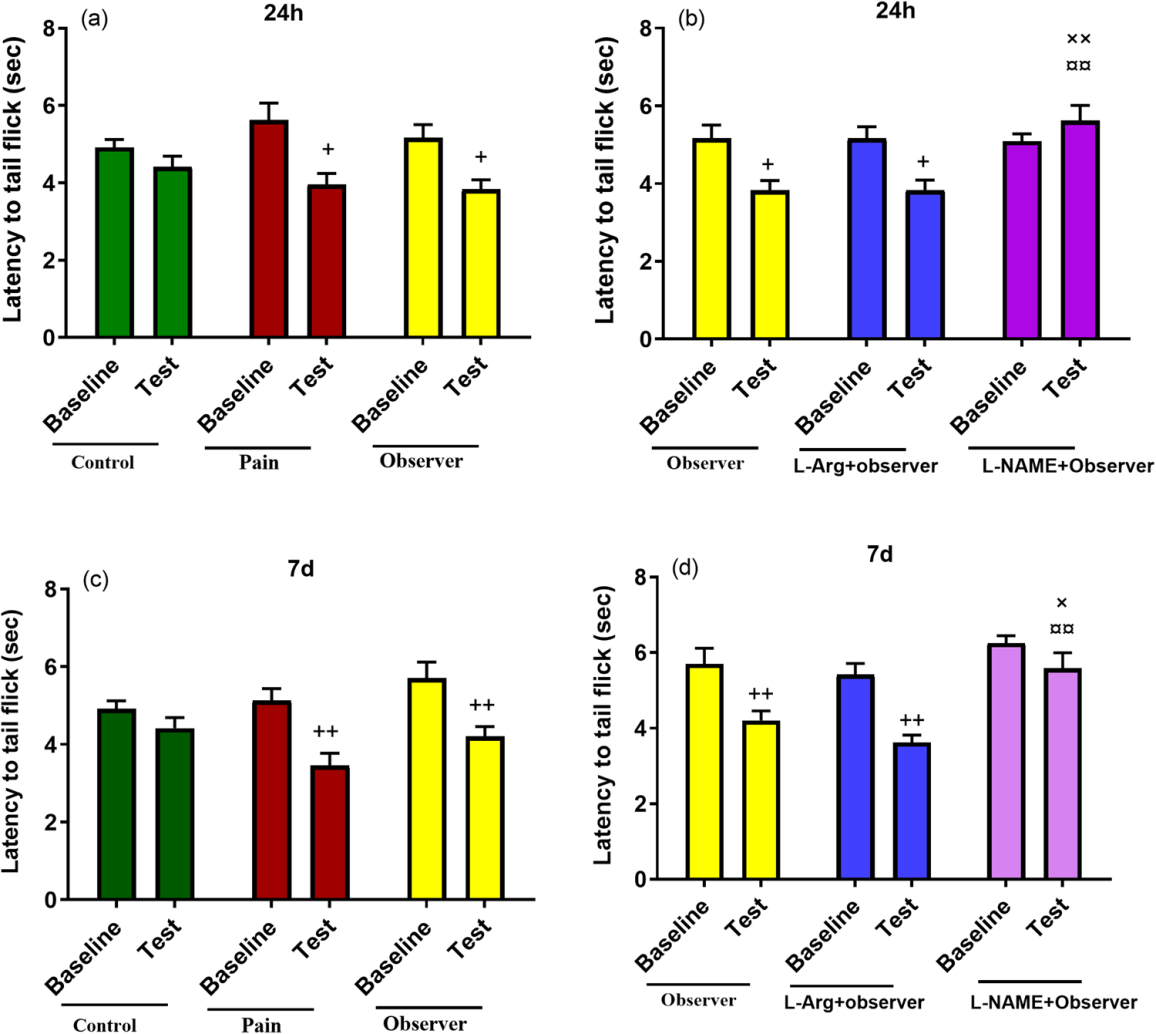
Effect of observation of sibling’s pain on tail flick latency in observer rats, 24 h (**a**) and 7d (**c**) after last observation. Comparison of the effects of L-Arg or L-NAME on the observers reaction time in the tail flick test (**b,d**). Results are shown as mean ± SEM. ^**+**^p < 0.05, ^**++**^p < 0.01, ^**×**^p < 0.05, ^**××**^p < 0.01, **¤¤**p < 0.01. ^**+**^, ^**×**^, **¤**, in comparison to baseline, observer and observer received L-Arg groups respectively.

In the tail flick test, the latency to a nociceptive response at 24 h and 7d after the last pain experience was significantly reduced in pain and observer groups in comparison to the pre-observation period (Fig. 2a, baseline & test: F_(1,42)_ = 21.6, p < 0.001, between groups: F_(2,42)_ = 0.4, p = 0.6; Fig. 2c, baseline & test: F_(1,42)_ = 24.5, p < 0.01, between groups: F_(2,42)_ = 2.43, p = 0.1), which provides further support of a hyperalgesic effect of empathic pain. Similar to findings in the hot plate test, the tail flick latency was significantly reduced in the L-arginine group, and L-NAME attenuated this effect (Fig. 2b, baseline & test: F_(1,42)_ = 6.8, p < 0.01, between groups: F_(2,42)_ = 4.4, p = 0.01; Fig. 2d, baseline & test: F_(1,42)_ = 27.3, p < 0.001, between groups: F_(2,42)_ = 10.7, p = 0.0002). The involvement of NO in nociceptive responses provides further support of the conclusion that NO is involved in empathetic pain induced by witnessing a siblings’ pain.

### NOx concentration in serum, 24 h and 7d after observation of siblings’ pain

**Figure 3:**
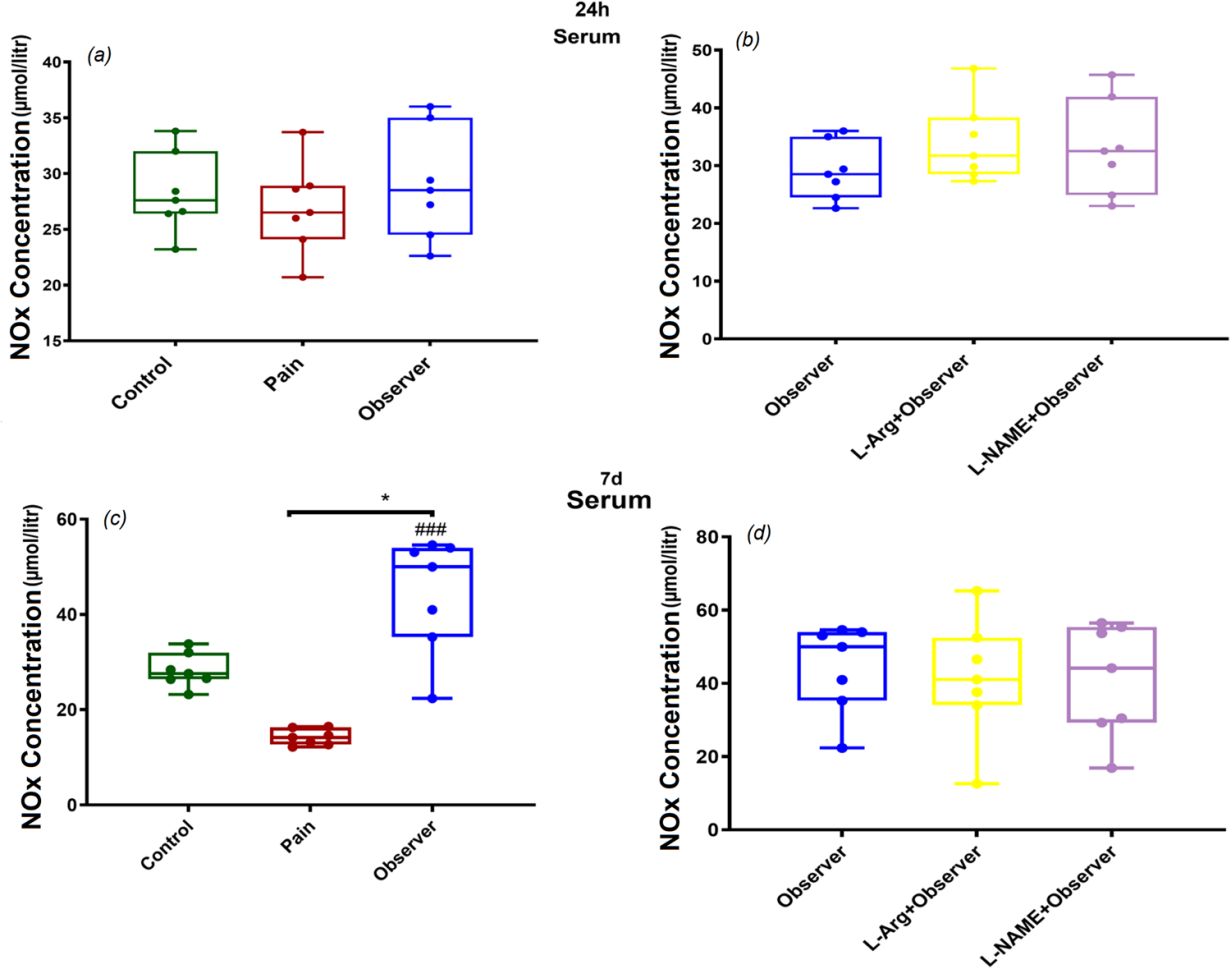
Effect of observation of sibling’s pain on NOx serum levels 24 h (**a,b**) and 7 days (**c,d**) after last observation. *p < 0.05, ###p < 0.001 in comparison to control and pain group, respectively. Results are shown as median with interquartile range (boxes) and maxima/minima (whiskers).

There were no significant differences in serum NOx concentrations between the observer group in comparison to control and pain groups 24 h after observation of a sibling in pain (Fig. 3a: F_(2,18)_ = 0.4, p = 0.6 and Fig. 3b: F_(2,18)_ = 1.02, p = 0.3). In contrast, 7d after the last observation, we noted higher NOx levels in the observer group compared to control and pain groups (Fig. 3c: F_(2,18)_ = 29.3, p = 0.001 and Fig. 3d: F_(2,18)_ = 0.1, p = 08). However, observers who were pretreated with L-NAME showed no significant changes in serum NO metabolites 24 h and 7d after the last observation.

### NOx concentration in left and right hippocampus, 24 h and 7d after observation of siblings’ pain

**Figure 4:**
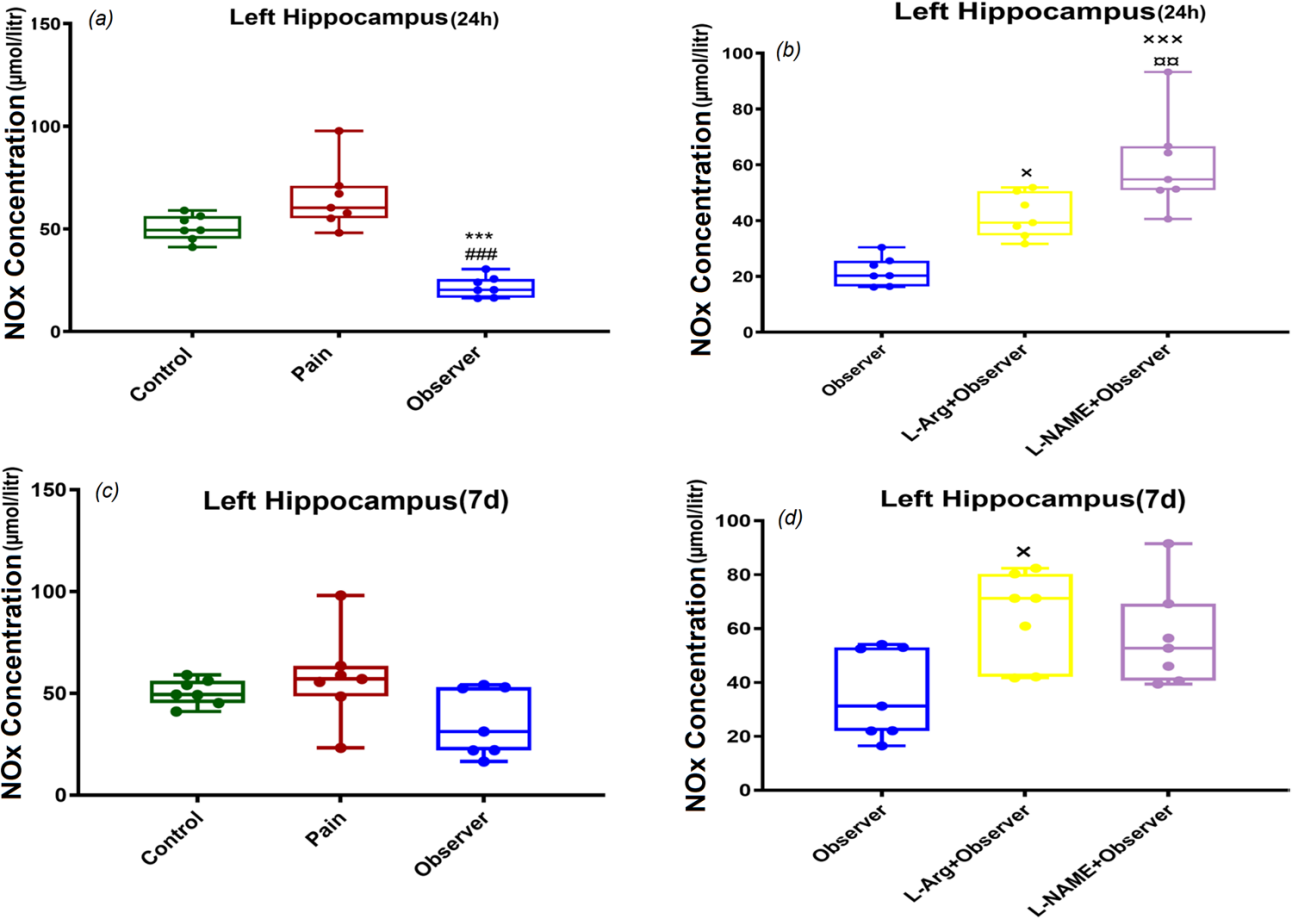
Effect of observation of siblings’ pain on NOx levels in left hippocampus 24 h and 7 days after last pain observation. ***p < 0.001 in comparison to control group, ^###^p < 0.001 in comparison to pain group, ^**×**^p < 0.05, ^**×××**^p < 0.001 in comparison to observer group and ^**¤¤**^p < 0.01 in comparison to observer recived L-Arg group. Results are shown as median with interquartile range (boxes) and maxima/minima (whiskers).

**Figure 5:**
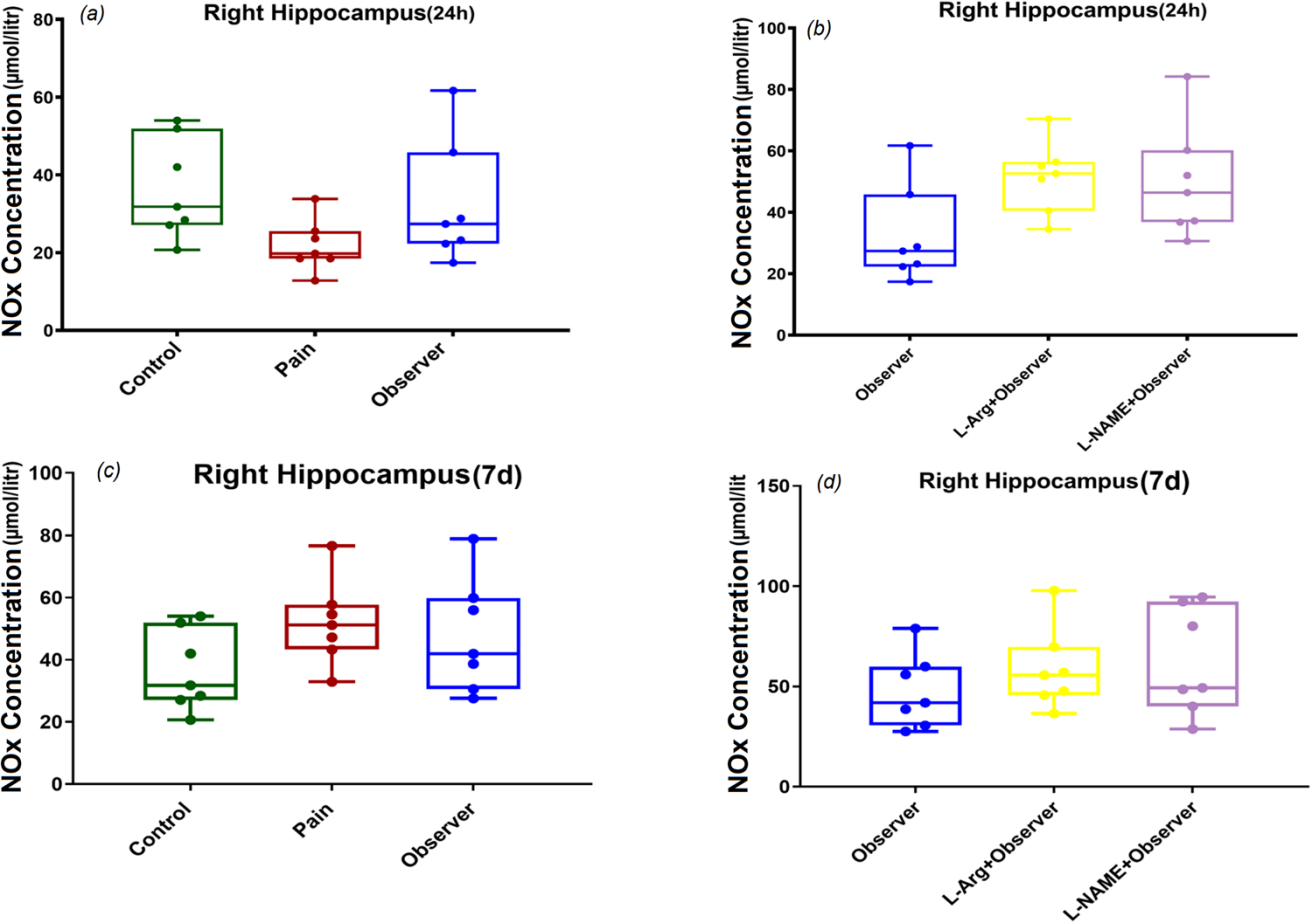
Effect of observation of siblings’ pain on NOx levels in right hippocampus 24 h and 7 days after last pain observation. Results are shown as median with interquartile range (boxes) and maxima/minima (whiskers).

Our data indicated that observation of sibling pain was associated with lower NOx levels in the left hippocampus than levels seen in control and pain groups 24 h (Fig. 4a, F_(2,18)_ = 31.3, p = 0.001) after the last pain observation. In addition, administration of L-Arg and L-NAME enhanced NOx values in the left hippocampus, 24 h after the last observation of sibling pain, however, the increase in NO metabolites was higher in observers who received L-NAME when compared with those receiving L-Arg (Fig. 4b, F_(2,18)_ = 20.5, p = 0.001). NOx concentrations were not significantly different 7d after the last observation in the left hippocampus (Fig. 4c, F_(2,18)_ = 3.2, p = 0.06), but pretreatment with L-Arg significantly increased NOx levels of left hippocampus in observer rats 7d after last observation (Fig. 4d, F_(2,18)_ = 4.9, p = 0.01). These data were in contrast to those noted in the right hippocampus as there  was no significant difference in NOx values in this region at either 24 h or 7d after the last pain observation (Fig. 5a: F_(2,18)_ = 2.6, p = 0.09, Fig. 5b: F_(2,18)_ = 3.2, p = 0.06, Fig. 5c: F_(2,18)_ = 1.9, p = 0.1, Fig. 5d: F_(2,18)_ = 0.8, p = 0.4).

### NOx concentration in cerebellum, 24 h and 7d after observation of sibling in pain

**Figure 6:**
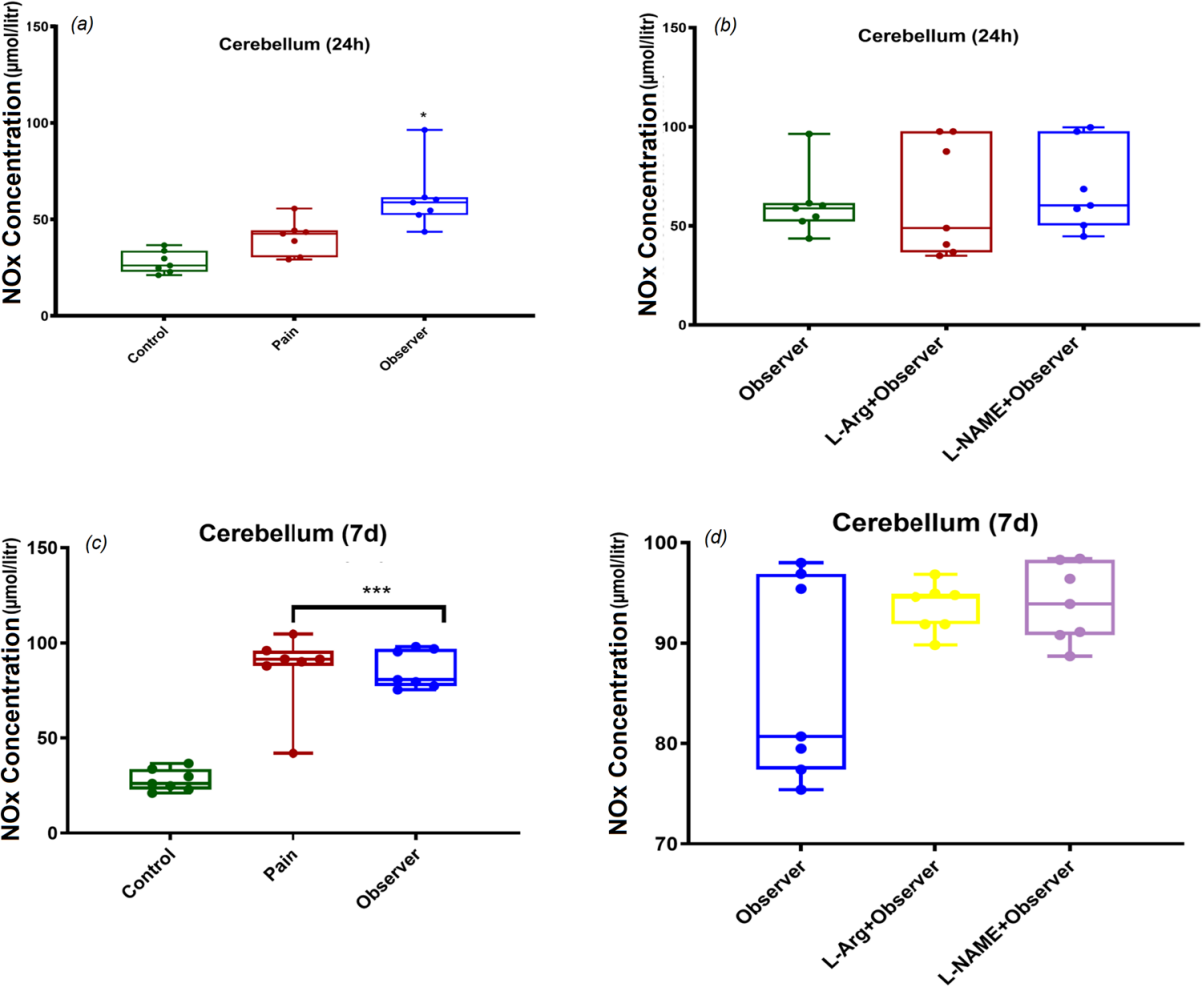
Effect of observation of sibling’s pain on cerebellum NOx levels in observer rats 24 h (**a,b**) and 7 days (**c,d**) after last observation. *p < 0.05 and ***p < 0.001 in comparison to control group. Results are shown as median with interquartile range (boxes) and maxima/minima (whiskers).

In the cerebellum, observer rats, displayed significantly higher NOx levels 24 h after the last observation than levels in control rats (Fig. 6a: F_(2,18)_ = 15.02, p = 0.001). A significantly higher NOx concentration was evident in pain and observer groups in comparison to control group 7d after the last pain observation (Fig. 6c: F_(2,18)_ = 43.8, p = 0.001). No effects of L-Arg and L-NAME on NOx levels were seen in this brain region (Fig. 6b: F_(2,18)_ = 0.1, p = 0.8 and Fig. 6d: F_(2,18)_ = 3.3, p = 0.6).

### NOx concentrations in prefrontal cortex, 24 h and 7d after observation of sibling in pain

**Figure 7:**
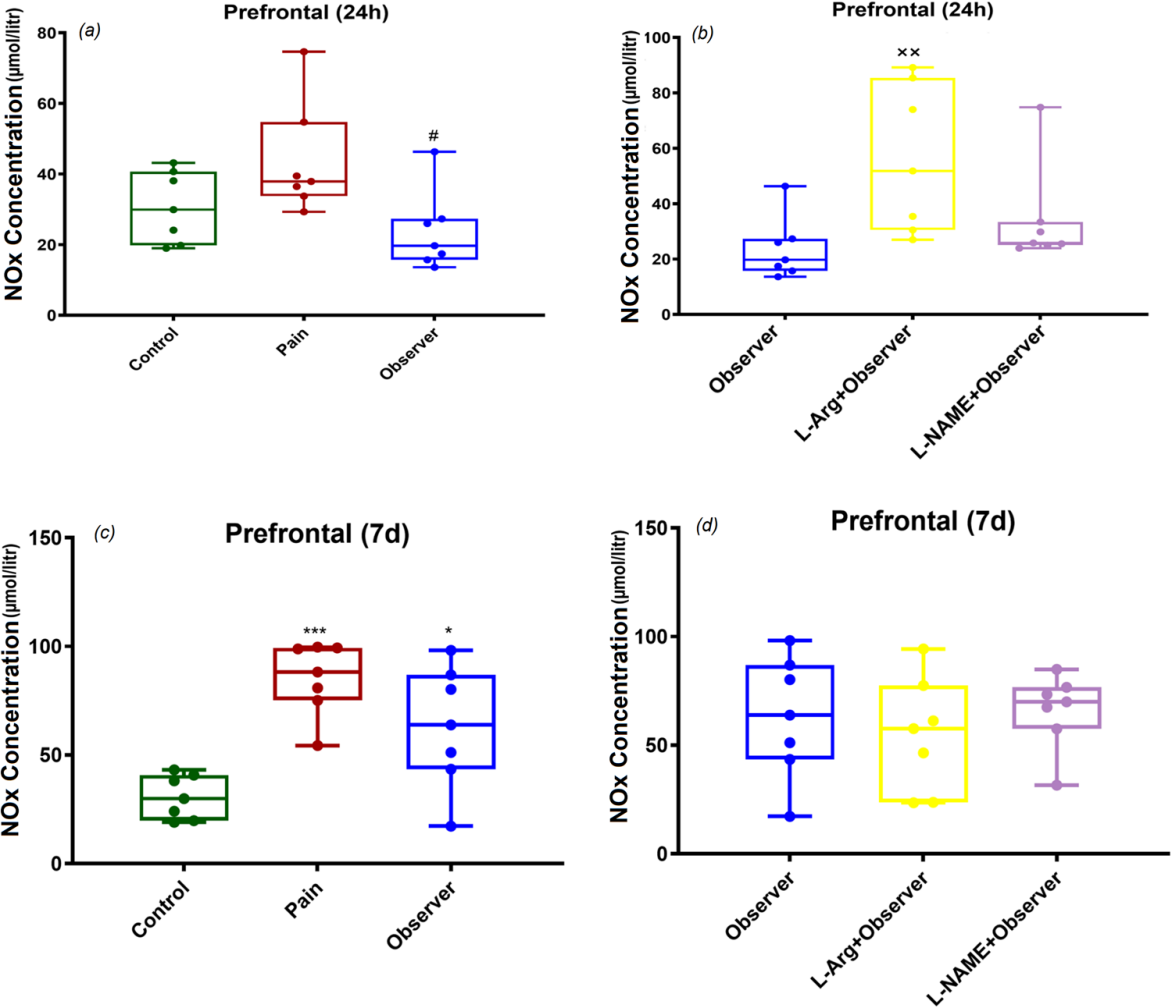
Comparison of the NOx levels in the control, pain, and pain observer groups in the mPFC. Compared to the pain group, 24 h after last observation, NOx values in observer group were lower (**a**) and L-Arg augmented the NOx level seen in the observer group (**b**). NOx level was significantly increased (**c**) 7 days after the last pain observation compared to control rats. In prefrontal cortex, administration of L-NAME had no effects upon NOx concentrations (**d**). *p < 0.05, ***p < 0.001 in comparison to control group, ^#^p < 0.05 in comparison to pain group, ^**××**^p < 0.01 in comparison to observer group. Results are shown as median with interquartile range (boxes) and maxima/minima (whiskers).

Within the prefrontal cortex, while lower levels of NOx concentrations were found in the observer group compared with levels noted in the pain group 24 h after observation of sibling pain (Fig. 7a: F_(2,18)_ = 4.5, p = 0.02), we didn’t find differences between observer rats and the control group in this parameter. Compared to the control group, 7 days after last observation, NOx values in pain and observer groups were higher (Fig. 7c: F_(2,18)_ = 13.5, p = 0.0003). L-Arg was found to be associated with higher NOx levels in the observer group 24 h (Fig. 7b: F_(2,18)_ = 4.9, p = 0.01) after the last observation, but there was no effect when analyzed at 7d (Fig. 7d: F_(2,18)_ = 0.4, P = 0.7) post observation. In prefrontal cortex, administration of L-NAME had no effect upon NOx concentrations.

## Discussion

Empathetic responses can result in social transmission of pain between familiar rats^2,3,5^. Previous studies have shown that observing a conspecific in pain increases the response of the pain-naïve rodent to a nociceptive stimulus^14,44^, including findings from our laboratory^42^. Further, we have shown that alterations in NO synthesis altered nociception in observers of conspecifics receiving pain^42^. Since NO plays an important role in pain^41^ and we have shown a role for NO in empathic pain^42^, the aim of this study was to further explore this role in brain regions involved in empathic pain. Conclusions from these earlier reports are supported by the current findings that a sibling’s pain decreased the nociceptive threshold in observer rats as assayed by a decreased reaction time in hot plate and tail flick testing at two different time points, 24 h and 7d, after the last pain observation. In the present study, we were able to replicate previous findings to show the existence of empathy in rats in response to a siblings’ pain and we extended them by showing NOx levels in brain regions involved in this activity.

A role for NO in thermal hypersensitivity induced by witnessing a conspecific's pain was shown by attenuation of this effect in presence of L-NAME. Further, we assayed NOx levels across 4 different regions of the rat brain to determine whether NO production increased or decreased in response to observation of pain in a sibling. Witnessing a pain experience in a sibling was associated with reduced NOx levels in the left hippocampus 24 h, but not at 7d, after the final observation. The cerebellum showed higher levels in observers at both 24 h and 7d after the last pain trial. Although several studies of the mechanisms underlying empathy have been conducted, our study presents novel data of  region- specific differences in NOx, which further supports a role of NO in empathetic responses generated in response to witnessing of pain. Further, our findings support earlier work^14^ showing that thermal hyperalgesia was induced uponwitnessing of a cagemate in pain, and extend those by showing a role for NO in the underlying mechanisms of empathy-related sensitization of nociception.

While studies in humans of empathetic pain can include subjective measures including self-reporting of pain, in rodents, indirect, objective measures of the pain experience must be utilized. We monitored alterations in the nociceptive threshold for thermal pain because in the pain experience, sensitization of the pain pathways occurs, which also results in lowering of this threshold, including to thermal pain^45^. In this study, observer and pain-experiencing rats exhibited lower nociceptive thresholds compared to the control groups at 24 h and 7d after the end of the pain experience. In line with our findings, Li *et al*., reported facilitated nociceptive responses when monitoring mechanical sensitivity in rodents observing pain experienced by cagemates^15^. However, in contrast to our findings, Li *et al*., did not observe thermal hypersensitivity, which was also in contrast to findings from Langford *et al*., who showed that thermal hyperalgesia was induced in mice who observed a cagemate in pain^14^. Differences were ascribed to different species in the two studies. However, it is of note that we have used two different tests reliant on thermal sensory processing, and in both cases, we noted hypersensitivity. Regardless, further, support of our data is provided by a recent report that observation of a conspecific experiencing a peripheral nerve injury was associated with an increase in the acetic acid–induced writhing test response in the observers^16^. In addition, mice who were co-housed with individuals who experienced CFA-induced hypersensitivity exhibited thermal and mechanical hyperalgesia^46^.

A link between NO and pain has been well established, however, the relationship is complex. At the level of the spinal cord, NO is an important neurotransmitter involved in pain transmission in sensory pathways (for review see, Cury *et al*.)^33^. Chronic administration of monosodium glutamate which stimulates NO-mediated neurotransmission pathway, attenuated the thermal nociceptive threshold and enhanced nociceptive behavior, which was associated with an increase in NOx levels^47^. Increased thermal and mechanical hyperalgesia can be induced in neuropathic rats upon i.p. injection of L-Arg. Further, NO was found to play a role in development of peripheral neuropathy in response to chronic sciatic nerve constriction^48^, whereas, i.p. administration, or direct application to the site of injury, of L-NAME resulted in decreased pain and thermal hyperalgesia in neuropathic rats, respectively^48,49^. NO regulates expression and/or activity of cyclooxygenases that increase prostaglandin E2 and prostacyclin which might lead to peripheral hyperalgesia, and pretreatment with L-NAME could impede these effects^50,51^. While many studies have shown that NO is involved in development of hyperalgesia, NO acting in the periphery or centrally can also participate in analgesia^52^. Interestingly, while high doses of L-Arg, presumably correlating with higher NO levels resulted in a greater perception to pain, lower concentrations were associated with analgesic effects which were attributed to attenuation of spinal cord pain transmission by inhibition of NMDA receptors and calcium channels^53^. Accordingly, NO transmission has dual effects in the periphery and centrally in mediation of pain, and the outcome could be dependent on local differences in levels of production.

While empathetic pain does result in activation of central pain pathways, because empathy does not directly stimulate peripheral sensory receptors, but rather relies on central cognitive processing involving social interaction and communication^54^, it is not clear that a role is played by NO in the physical manifestation of empathetic pain. However, a link has been suggested. In male criminal offenders with a variant of the nNOS gene^41^, an association was found between this gene with impulsiveness and empathy, suggesting that the nNOS gene product plays a regulatory role in social behaviors including empathy^41^. In an earlier study, we found that changes in the NO system modulated effects on empathetic pain^42^. We extend those findings by reporting here that pretreatment with L-NAME attenuated the hyperalgesia effect in the observer group, and L-Arg resulted in reducing this effect, demonstrating that NO plays a role in manifestation of pain empathy. Further, we found evidence of alterations in NO metabolism. Due to the short half-life of nitric oxide, direct measurement of NO is difficult, therefore, we measured metabolites of NO (NOx) since changes in the levels of these inactive compounds have been successfully demonstrated in biological tissues^55^. When we evaluated NOx levels in serum, we found that while levels were not affected 24 h after the last pain observation, when compared to both controls and those experiencing pain, they were elevated in observers 7d later. The difference seen in observers between 24 h and 7d may have been due to the time required for metabolism and breakdown of NO, which relies on many factors and can vary substantially. Interestingly, the significant difference in NOx levels in serum of observers at 7d which was not an effect seen in those experiencing pain suggests differences in the processes underlying formation or degradation of NO in empathetic pain when compared to those involved in the physical experience of pain.

The experience of pain activates a pain matrix which is composed of affective and sensory pathways^56^. Vachon-Presseau *et al*., indicated that the observation of pain also appears to activate both pathways^57^. Human imaging studies have shown that similar brain regions can be activated in subjects who experience physical pain first-hand and in individuals who observe physical pain being experienced by a loved one^58–60^. Common brain regions activated by first-hand pain and observance of pain are the rostral anterior cingulate cortex (ACC), bilateral anterior insula (AI), cerebellum and brainstem^58–60^. In rodents, electrophysiological studies have shown that neurons in the ACC can respond both to first-hand experiences of pain and observation of pain^29^. The ACC is included within the mPFC^28^, and in rodents, the mPFC was shown to play a crucial role in production of empathy for pain as chemical lesion of the mPFC^13,60^ abolished the empathetic response to mechanical pain. Precision lesions of the ACC are difficult in rodents, and determination of the exact region of the mPFC involved in empathetic pain was not provided, however, the role of the mPFC in emotional control suggested existence of a top-down processing phenomenon in production of empathy for pain^13,60^. Although the hippocampus has rarely been examined for empathy, a previous study showed that patients with hippocampal lesions exhibited reduced empathy and prosocial behavior^31^.

Our evaluation of alterations in NO in neural regions shown to be activated in other studies of empathetic pain revealed region and time-specific differences. Rats witnessing a painful experience in a sibling demonstrated reduced levels of NOx in the left hippocampus 24 h after the last observation when compared to levels seen in the control and pain groups. In contrast, there were no differences seen in the right hippocampus 24 h after the last observation. Further, the left and right hippocampus did not show any changes in NOx levels 7d after the last observation of pain experienced by a sibling. We also found that treatment with L-Arg enhanced NOx levels in the left hippocampus of the observer group 24 h and 7d after the end of observation. Further, administration of L-NAME was associated with increased NOx values in the observer group 24 h after last observation and the enhancement induced by L-NAME was greater than that seen in those receiving L-Arg. This latter finding is similar to that seen by Miller *et al*., who showed that sustained treatment with L-NAME (100 mg/kg) for 7d resulted in elevated NO concentrations. NO production was reported to be insensitive to effects of L-NAME which was suggested to be due to compensatory mechanisms involving upregulation of specific isoforms of NOS triggered by suppression of the NO system^61^. While we did not explore the mechanisms underlying the reduction in hippocampal NOx associated with empathetic pain, the effect might have been due, in part, to changes in activity of NOS isoforms, as this would result in alterations in the level of NO metabolites.

Since the strong lateralization of hippocampal functions has been proved (for review see., Jordan 2019)^32^, based on results of our study, left hippocampus maybe exhibit striking functional lateralization in empathic pain, however, electron paramagnetic resonance imaging techniques are needed to clarify the distribution of the NOX in hippocampi.

In our study, we found an increase in NOx in cerebellum 24 h and 7d after witnessing of pain, and higher levels were also found in PFC after the last observation of siblings’ pain which were changes similar to those seen in the pain group. Pretreatment with L-NAME or L-Arg had no effect on NOx levels in these areas. While there are no data to support a role of NO in the cerebellum or brain stem in empathetic pain, our findings are supported by studies showing enhanced NOx levels in these two brain regions in rats experiencing neuropathic pain, and decreases in NOx levels in cerebellum resulted in attenuation of neuropathic pain in these subjects^62^. Supporting the hypothesis that alterations in NOS could be mediated by cognitive processes such as empathetic pain, signal for nNOS was higher in mPFC in mice exposed to predator stress^63^. Although we did not examine the mechanisms underlying rises in NO, the increase in NOx levels could be due to activation of NMDA receptor- mediated neuronal nitric oxide synthase pathway which plays key role in the negative affective component of pain^64^.

As a limitation of our study, experiments were restricted to male rats. Several lines of evidence suggest that there are significant sex differences in the display of empathy, with females demonstrating higher levels of affective and sympathetic behaviors, including empathy^65,66^. However, it is not known whether sex differences observed in human empathy extend to the animal. Using a rat model of emotional contagion, Mikosz *et al*., (2015) compared the behavioral consequences of social transfer of information in male and female rats and found sex, and estrus cycle phase differences in susceptibility to emotional transfer^24^. Further, the estrus cycle influences metabolism of NO^67^. To eliminate the possible influence of gender on obscuring results, in the current study, only male animals were used. As pain and empathy show sex-based differences, further studies on female ovarectomized rats or in females during different phases of the menstrual cycle  are warranted to evaluate the role of sex hormones on NOx expression associated with empathetic pain.

In conclusion, our results suggest that NO may be involved in development of empathic thermal hyperalgesia and further, that observation of sibling’s pain can change metabolites of NO in different brain regions of observer rats. Clearly, conflicting results on the levels of NOX in different regions make interpretation difficult, and comprehensive experiments are needed in order to elucidate and to pinpoint the mechanisms behind region-specific differences in NO metabolism associated with witnessing of pain in others, and the exact role of NO in empathic pain.

## Material and Methods

### Animals

**Figure 8:**
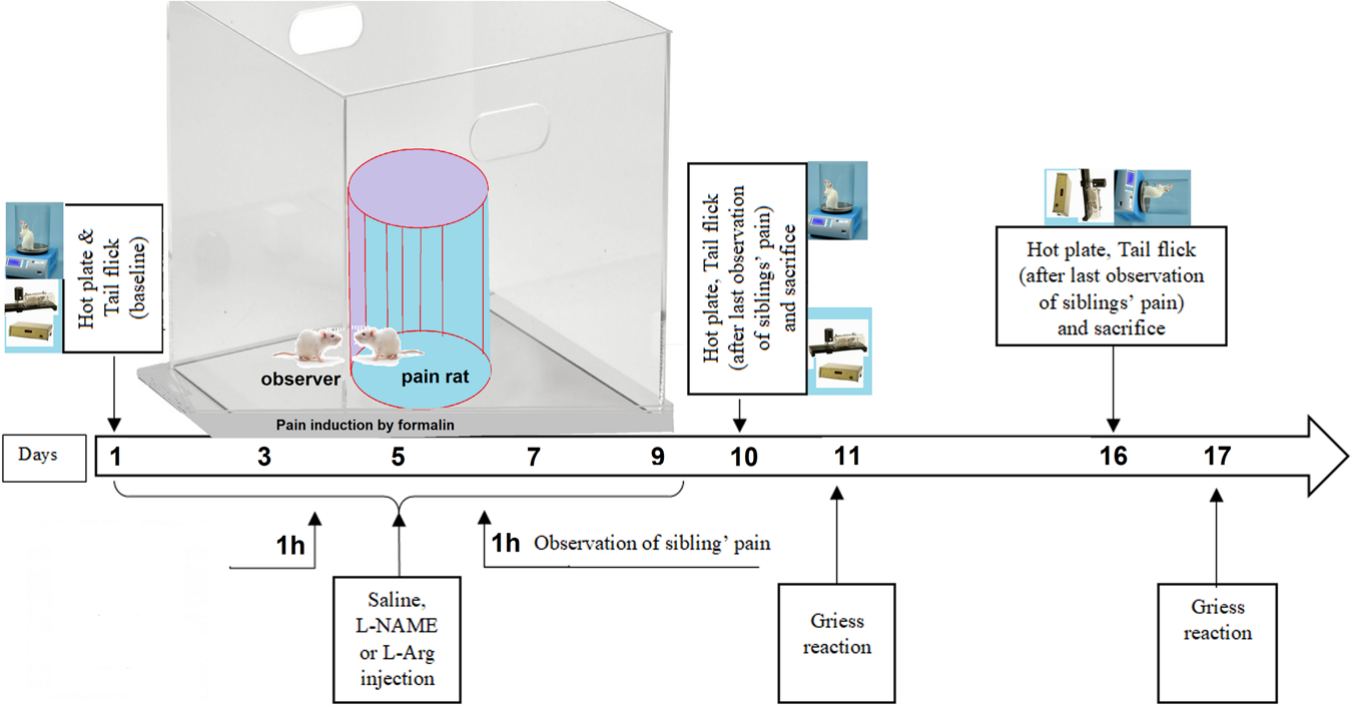
Timeline for L-Arg and L-NAME administration, formalin injection, observation of siblings’ pain, behavioral procedures and Greiss method.

Female and male Wistar rats were purchased from the Kerman University of Medical Sciences (weighing 220–280 g) and were used as breeders. They were housed in a room with standard temperature (22 ± 2 °C) and 12/12 light dark cycle (lights on: 07:00–19:00 h). All the procedures were approved by the Kerman University of Medical Sciences Ethic Committee (IR.KMU.REC.1397.342). Seventy male offspring rats (weighting 150–180 g) were divided into five groups: Control, pain (with formalin injection), observer (observation of siblings’ pain), observer +L-Arginine (a precursor of NO) and observer+L-NAME (L-arginine methyl ester dihydrochloride; a nonspecific NO synthase inhibitor). Animals in each group were placed two by two in the cage. In this study, we designed an animal model of empathy by sub-chronic observation of siblings’ pain which is somewhat similar to human life. On the first day of study, one animal was selected as the observer and another was exposed to the painful stimuli, which consisted of a formalin injection (50 µL, 10%) with an insulin syringe into the plantar surface of the hindpaw. Following this, the injected rat was placed beside the observer in the open field box (Fig. 8, timeline procedure). After each witnessing of siblings’ pain, rats were separated, but for the prevention of the destructive effects of isolation, the demonstrator rats were placed in one cage and the observer rats were placed in the other cage (rats housed 2 per cage according to RAT HOUSING GUIDELINES 2016 (University of California, Berkeley) based on the work of Cox *et al*., which supports the idea that even if observer and demonstrator were not physically together, the observer would experience empathy^68^. This procedure was repeated every other day for nine days. Therefore, each observer observed their sibling’s pain upon a total of 5 formalin injections. In order to reduce tissue damage caused by formalin, injections were alternated between the right and left hind paws, which provided an injection interval of 4 days for each paw. Observers in each group received saline, L-Arginine or L-NAME (Sigma-Aldrich, UK) (10 mg/kg, i.p.) 1 h before observation of pain. Pain sensitivity was evaluated using hot plate and tail flick tests in pre and post-observations. For nitrate and nitrite (NOx) assays in blood sample and brain tissue, animals scarified on the tenth and sixteenth day after the first day of the pain observation.

All experiments were performed in accordance with relevant guidelines and regulations.

### Nociceptive response assessment

Pain threshold pre (baseline) and post observation of sibling’ pain was assayed using procedures previously published^42,69,70^. Briefly, a hot plate apparatus made of a plate with a diameter of 19 cm and a plexiglass wall of 30 cm height was heated to a temperature of 52 ± 0.5 °C. The reaction time after placing the animal on the plate was recorded for each animal with the latency to reaction being the time to the first observation of licking a hind-paw or jumping. Using this method, an animal was removed after 45 seconds to avoid tissue damage.

Tail flick was used for assessment of the response to acute thermal stimuli applied to the tail region. Flicking of the tail is a reflex-protective response demonstrating the responsiveness of neurons at the spinal level. Animals were restrained in a restrainer and their tails were free and hanging outside the restrainer. The lower 5 cm portion of the tail was marked and placed under an intense light, and the period between start of light exposure and the observation of tail movement was recorded for each animal^71^.

### Serum separation

After euthanization with CO_2_, animals were decapitated and trunk blood was collected in plastic centrifuge tubes and centrifuged at 4000 rpm for 15 min. After that, serum was collected into the micro tubes and was kept in −80 °C till the time of assay.

### Brain tissue separation

Animals were sacrificed and after blood sampling, the brain was immediately removed. The mPFC, left and right hippocampus and cerebellum tissues were carefully dissected. After dissection, the 4 regions of the brain were stored in liquid nitrogen and refrigerated at −80 °C until they were analyzed.

### Measurement of nitrate and nitrite (NOx) levels in serum and brain tissues

Serum and tissue NOx levels were measured using the Griess method^37^. In brief, tissues were unfrozen and homogenized in PBS (1:5, w/v), then serum and tissues were centrifuged at 10,000 g for 10 min and 10,000 g for 15 min respectively. The supernatants of samples were deproteinized by adding 15 mg/ml zinc sulfate, then samples were recentrifuged at 15,000 g for 20 min. In both cases, 100 μL of the clear supernatants were transferred to a 96 microplate well and 100 μL vanadium (III) chloride (8 mg/ml) was added to each well to reduce nitrate to nitrite. Thereafter, 50 μL of Griess reagents (sulfanilamide (2%) and N-1-(naphthyl) ethylenediamine (0.1%)) were added to each well and the mixture was incubated for 30 min at 37 °C. The absorbance values in each well were measured using the ELISA reader at 540 nm wavelength (BioTek, Powerwave XS2,). Concentration of NOx in samples was calculated from the linear standard curve established by 0–100 μM sodium nitrate. Tissue NOx levels were expressed as μmol/L. Inter-assay coefficient of variation was 4.2%^72^.

### Statistical analysis

The Kolmogorov-Smirnov test was used to determine the normal distribution of the data. Normally distributed data  were compared by one-way or two-way ANOVA and the Tukey post hoc analysis was used for multiple comparisons. A two-way ANOVA was used to compare nociceptive responses among all groups in pre and post observations. Data that was not normally distributed was analyzed using nonparametric tests (*Kruskal-Wallis or Friedman*). Data with normal distribution are expressed as mean ± SEM and nonparametric data are presented as median and interquartile range. P < 0.05 (two-sided) was considered statistically significant between groups.
